# Announcements

**Published:** 2015-05-01

## Amyotrophic Lateral Sclerosis (ALS) Awareness Month — May 2015

May is Amyotrophic Lateral Sclerosis (ALS) Awareness Month. ALS, also known as Lou Gehrig’s disease, is a progressive, fatal, neurodegenerative disorder of the upper and lower motor neurons. The etiology of ALS is not well understood, and no cure exists. Persons with ALS usually die within 2–5 years of diagnosis.

In October 2010, the Agency for Toxic Substances and Disease Registry (ATSDR) launched the congressionally mandated National ALS Registry (https://wwwn.cdc.gov/als/Default.aspx) to collect and analyze data regarding persons with ALS in the United States. The goals are to determine the incidence and prevalence of ALS, characterize the demographics of those living with ALS, and examine potential risk factors for the disease. ATSDR released the first National ALS Registry report in July 2014 for persons living with ALS in the United States during October 19, 2010–December 31, 2011 (1). Findings in this report indicated that approximately 12,000 people were identified with ALS during this period, or approximately four in every 100,000 persons. ALS is more common in whites, males, non-Hispanics, and persons aged 60–69 years. These findings are consistent with well-established European ALS registries and small epidemiologic studies that have been conducted in the United States.

ALS, like most noninfectious diseases, is not a notifiable disease in the United States. To collect data on cases, the registry uses data from existing national databases, including the Centers for Medicare and Medicaid Services and the U.S. Department of Veterans Affairs, as well as information provided by persons with ALS through the secure online system. Online registrants also can take brief surveys regarding potential risk factors for the disease (e.g., occupational, military, smoking, alcohol, and residential histories).

ATSDR is collaborating with the ALS Association (http://www.alsa.org), Muscular Dystrophy Association (http://www.als-mda.org), Les Turner ALS Foundation (http://www.lesturnerals.org), and other organizations to make all persons with ALS and their families aware of the opportunity to register in the National ALS Registry. Additional features have been added to enhance the registry for patients and researchers, including state and metropolitan area–based ALS surveillance to assist in evaluating the completeness of the registry and to provide local incidence and prevalence data, a research notification system to inform persons with ALS about new research studies, a biorepository study to evaluate the feasibility of collecting biospecimens from enrollees, and mobile apps to help find the nearest ALS clinics and support groups.
